# The Hippo signalling pathway and its impact on eye diseases

**DOI:** 10.1111/jcmm.18300

**Published:** 2024-04-13

**Authors:** Yuxiang Du

**Affiliations:** ^1^ Precision Medicine Laboratory for Chronic Non‐communicable Diseases of Shandong Province, Institute of Precision Medicine Jining Medical University Jining Shandong People's Republic of China

**Keywords:** eye disease, Hippo, TAZ, therapy, YAP

## Abstract

The Hippo signalling pathway, an evolutionarily conserved kinase cascade, has been shown to be crucial for cell fate determination, homeostasis and tissue regeneration. Recent experimental and clinical studies have demonstrated that the Hippo signalling pathway is involved in the pathophysiology of ocular diseases. This article provides the first systematic review of studies on the regulatory and functional roles of mammalian Hippo signalling systems in eye diseases. More comprehensive studies on this pathway are required for a better understanding of the pathophysiology of eye diseases and the development of effective therapies.

## 1 INTRODUCTION

The Hippo signalling pathway is an evolutionarily conserved pathway that plays an integral role in regulating glucose metabolism,^1^ cellular proliferation, fate regulation, apoptosis, and control of tissue growth and regeneration.^2^ The key effector proteins of the mammalian Hippo signalling pathway, yes‐associated protein (YAP) and transcriptional coactivator with a PDZ‐binding motif (TAZ), collectively known as YAP/TAZ, regulate cell fate decisions.^3^ Activation of the canonical Hippo pathway leads to the formation of the sterility 20‐like protein kinase (MST1/2) and Salvador 1 (SAV1) complex, which subsequently phosphorylates the large tumour suppressor (LATS1/2) and MOB kinase activator 1 (MOB1). The activated LATS1/2‐MOB1 complex, in turn, phosphorylates YAP/TAZ, resulting in YAP/TAZ retention and degradation in the cytoplasm, thereby preventing their accumulation in the nucleus and downstream gene expression.^4^ In addition to the canonical Hippo pathway, the MST1/2‐SAV1‐LATS1/2‐MOB1‐YAP/TAZ axis, other factors such as neurofibromin 2 (NF2),^5^ mitogen‐activated protein kinase kinase kinase kinases (MAP4Ks)^6^ and nuclear Dbf2‐related1/2 (NDR1/2)^7^ are also identified thus enriching our understanding regarding the Hippo pathway.

On the other hand, when the Hippo pathway is inactivated, dephosphorylated YAP/TAZ enters the nucleus where they interact with other transcription factors to regulate downstream gene transcription.^8^ Since YAP/TAZ lack DNA‐binding sites, they exert their biological effects by forming complexes with various transcription factors, including TEA domain DNA‐binding family members (TEAD1‐4), SMADs and the Runt‐related transcription factor (RUNX) family.^9^, ^10^ The expression of downstream target genes of the Hippo pathway, such as connective tissue growth factor (CTGF),^11^ cysteine‐rich angiogenic inducer 61 (CYR61),^11^ ankyrin repeat domain 1 (ANKRD1),^12^ and MYC proto‐oncogene bHLH transcription factor (MYC),^13^ regulates cell proliferation and survival.

The Hippo signalling system has been demonstrated to be critical in embryonic development. Homozygosity for the Yap allele results in developmental arrest in mice around E8.5.^14^ It is also essential for eye development.^15^ Its involvement in multiple aspects of embryonic eye development, including lens development,^16^ retinogenesis,^17^ retinal vascular development and barrier function,^18^ has been observed. Conditional ablation of YAP results in coloboma in the developing mouse eye.^19^ Heterozygous loss‐of‐function mutations in YAP lead to optic fissure closure defects during development, resulting in congenital malformations of the eye in patients.^20^ In addition, Fossdal et al. identified a point mutation in TEAD1 (Y421H) that affected the interaction between TEAD1 and YAP, resulting in Sveinsson chorioretinal atrophy, a hereditary ocular disease.^21^

**FIGURE 1:**
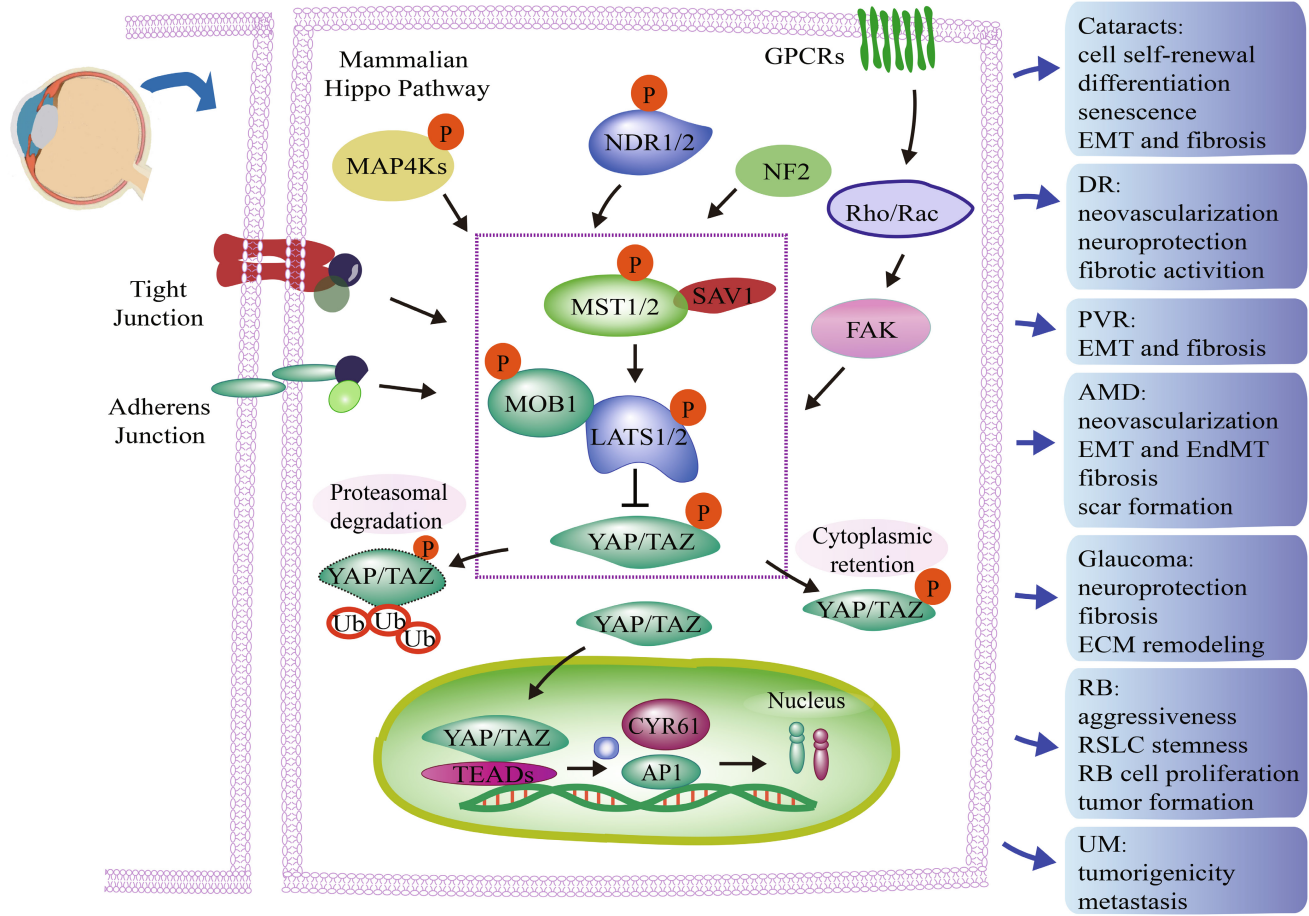
Functions of the Hippo pathway in mammalian eye diseases. Summary of eye diseases caused by dysregulation of the Hippo pathway. The components within the dotted rectangle indicate components of the canonical kinase cascade. Dysregulation of the Hippo pathway has been found in a variety of eye diseases and is involved in the regulation of the occurrence or progression of these diseases. Specific functions are shown in boxes. MAP4Ks, mitogen‐activated protein kinase kinase kinase kinases; NDR1/2, nuclear Dbf2‐related1/2; NF2, neurofibromin 2; GPCRs, G protein‐coupled receptors; FAK, focal adhesion kinase; MST1/2, sterility 20‐like protein kinase1/2; SAV1, Salvador 1; MOB1, MOB kinase activator 1; LATS1/2, large tumour suppressor 1/2; YAP, yes‐associated protein; TAZ, transcriptional coactivator with a PDZ‐binding motif; TEADs, TEA domain DNA‐binding family members; CTGF, connective tissue growth factor; AP‐1, activator protein 1; DR, diabetic retinopathy; PVR, proliferative vitreoretinopathy; AMD, age‐related macular degeneration; RB, retinoblastoma; UM, uveal melanoma; EMT, epithelial‐mesenchymal transition; EndMT, endothelial‐mesenchymal transition; ECM, extracellular matrix; RSLC, retinoblastoma stem‐like cells.

Numerous studies have also revealed that this cascade has a significant impact on several eye conditions including cataracts,^22^ diabetic retinopathy (DR),^23^ age‐related macular degeneration (AMD),^24^ proliferative vitreoretinopathy (PVR),^25^ retinoblastoma (RB)^26^ and uveal melanoma (UM).^27^ In this review, recent advances in the association between the Hippo pathway centered on YAP/TAZ, the ocular system and common ocular diseases are outlined (Figure 1). Furthermore, the involvement of ocular pathogenesis and the corresponding treatments have been highlighted. This is the first systematic review of the current understanding of the regulation and function of the Hippo pathway in ocular diseases, highlighting the need for further research in this field.

## 2 HIPPO SIGNALLING PATHWAY IN EYE DISEASES

### 2.1 Cataracts

Cataracts are a primary cause of blindness, particularly in developing countries. Based on the location of opacification within the lens, cataracts can be divided into three types: nuclear, cortical and posterior subcapsular cataracts.^28^ Currently, surgery is the only effective treatment for cataracts. To effectively restore vision, the cataractous lens was replaced with a man‐made plastic lens.^29^ Lens epithelial cells (LECs) are the source of clear avascular ocular lenses. LECs migrate to the lens equator and form lens fibres, which gradually condense and compress, leading to lens nucleus hardening and opacity.^30^ Loss of LEC homeostasis, abnormal proliferation and posterior displacement can all contribute to cataracts and posterior capsular opacification (PCO).^31^, ^32^

According to previous studies, YAP is essential for self‐renewal and differentiation of lens progenitor cells. It also maintains LEC shape by stabilizing junctional proteins and apical polarity complexes.^33^ In addition, YAP is necessary to preserve the normal size of the lens.^34^ The lens epithelium was significantly reduced in mice with conditionally deleted YAP.^33^ Loss of YAP inhibits proliferation, promotes abnormal differentiation and senescence of LECs, and eventually leads to an abnormal lens structure and nuclear cataract formation.^22^ He et al. showed that conditional YAP deletion in mice resulted in severe nuclear cataracts with an incidence rate of 98.2% at three months.^22^ Recent studies have demonstrated that, in mice, one allelic deletion of *Yap1* dramatically lowered LEC cell density, disrupted local cell adhesion and caused cataracts.^34^, ^35^ Liu et al. showed that YAP and glucose transporter 1 (GLUT1) protein levels were downregulated in LECs from old mice and age‐related cataract patients, whereas MST2 and phosphorylated YAP protein levels were upregulated. It has been suggested that MST2 and YAP are involved in oxidative stress‐induced apoptosis of LEC, and blocking the MST2/YAP/GLUT1 signalling pathway may prevent age‐related cataracts.^36^ YAP activation mediates NF2‐related pathology. YAP activation in patients with NF2 often leads to presenile cataract formation.^37^ *Yap* heterozygosity suppresses cataracts induced by *Nf2* deletion, in mice.^38^

PCO, the most common complication of cataract surgery, leads to visual disturbances and a decreased quality of vision.^39^ This is mainly due to LECs' differentiation to fibre cells for lens regeneration or transdifferentiation to myofibroblasts.^39^ Research has shown that YAP/TAZ also play an important role in lens regeneration. Li et al. found that the autophagy‐associated pathway regulates the YAP pathway by upregulating and secreting HSP90, thereby promoting the differentiation of LECs into fibroblasts and inducing PCO.^40^ Transforming growth factor‐β (TGF‐β) signalling is also activated after cataract surgery, which can induce epithelial‐mesenchymal transition (EMT) and fibrosis through the YAP signalling pathway in the PCO.^39^ Many factors, such as calponin,^41^ fibroblast growth factor,^16^ and mechanotransduction of stress,^42^ can promote cell differentiation by activating the YAP/TAZ axis and converting LECs into fibrous cells. Although the role of the Hippo/YAP pathway in cataracts remains unknown, targeting of YAP/TAZ appears to be a viable treatment option.

### 2.2 DR

DR is a common complication of diabetes that leads to progressive loss of vision and blindness. Clinically, DR is divided into two different stages: the initial stage, known as non‐proliferative DR and the progression to neovascularization, known as proliferative DR.^43^ Studies on the role of the Hippo pathway in DR have primarily focused on the proliferative DR stage. Angiogenesis and endothelial cell (EC) proliferation are the major pathogenic symptoms of DR, and are characterized by retinal microvascular anomalies and neurodegeneration.^23^ Although several treatment methods have been employed in clinical settings, their therapeutic efficacy is far from satisfactory.^44^ Therefore, it is critical to clarify the precise molecular aetiology of DR to identify ways to prevent its progression. Previous studies have shown that DR may be influenced by the Hippo pathway. For example, gene set enrichment research based on RNA sequencing analysis of patients with DR highlights the Hippo signalling pathway.^44^ Additionally, transcriptomic analysis of human post‐mortem retinal samples linked differentially expressed transcripts associated with late‐stage DR to the Hippo signalling pathway.^45^

During DR progression, many factors, including circular RNA^46^ and hyperglycemia,^47^ can cause retinal vascular dysfunction and promote endothelial angiogenesis via YAP/TAZ. Hippo pathway factors such as LATS^48^ and YAP/TAZ^49^ have been reported to be involved in endothelial cell proliferation, sprouting, tube formation and angiogenesis.^23^ Additionally, the Hippo signalling pathway regulates neovascularization in DR by interacting with the VEGF signalling pathway. YAP is highly expressed in the retinas of DR mice and promotes the proliferation and migration of retinal microvascular ECs by upregulating the expression of metastasis‐associated lung adenocarcinoma transcript 1 (MALAT1) and VEGFA.^50^, ^51^ On the other hand, the VEGF signalling pathway also regulates the Hippo signalling pathway. VEGF receptor activation inhibits LATS and activates YAP/TAZ to regulate VEGF‐induced retinal angiogenesis.^48^ Several medications, such as the Chinese patent medicine Compound Xueshuantong Capsule,^23^, ^52^ folic acid^47^ and glucocorticoids,^53^, ^54^ reverse retinal vascular dysfunction by inhibiting the YAP/TAZ pathway, thereby ameliorating diabetic macular edema and reducing the incidence of complications. These results suggest that YAP/TAZ may be a potential therapeutic target for DR.

In addition to its role in neovascularization‐associated ECs, the Hippo signalling pathway is implicated in the control of retinal Müller cells and retinal pigment epithelial (RPE) cells in the progression of DR. Retinal Müller cells are the major macroglia in the retina that contribute to neuroprotection during DR. Dephosphorylated YAP enhances the proliferation and activation of retinal Müller cells, thereby alleviating DR development by promoting glutamine synthetase.^55^ However, in the late stages of DR, YAP promotes fibrotic activity of Müller cells, leading to diabetes‐induced retinal fibrosis, haemorrhage and retinal detachment.^56^, ^57^ Degeneration or dysfunction of human RPE cells has also been shown to contribute to the pathological progression of DR.^58^ YAP upregulation may promote apoptosis, inflammation and oxidative stress in human RPE cells induced by high glucose and circular RNAs, thereby promoting the development of DR.^59^ DR amelioration may be attributed to the inactivation of the YAP pathway in rats.^60^ These results imply that the Hippo signalling pathway is crucial for the development of DR and holds promise as a targeted therapy for the prevention of DR.

### 2.3 PVR

PVR often occurs after corrective surgery for retinal detachment or posterior segmental trauma. PVR is a potential blinding complication of ocular fibrosis, and the EMT of RPE cells is the key pathological mechanism of PVR.^61^ PVR develops through a complex cellular process characterized by membrane proliferation, gliosis and contraction on or beneath the retina.^62^ RPE cells have long been recognized as important players in the pathophysiology of PVR, even though the epiretinal membrane contains various cell types. RPE cells undergoing EMT exhibit significantly enhanced proliferation and migration capabilities, which are the hallmarks of PVR.^63^

Disruption of cell junction complexes in RPE cells caused by various reasons can lead to EMT, retinal fibrosis and PVR by activating signalling pathways, such as Wnt and Hippo signaling.^64^, ^65^ Adherens junction and tight junction complexes promote YAP/TAZ phosphorylation and inhibit YAP/TAZ translocation into the nucleus.^66^ Loss of cell–cell contact results in TAZ nuclear translocation, activation of TAZ‐TEAD1 binding, ZEB1 expression and EMT. TAZ knockdown inhibits proliferation and EMT in primary mouse RPE cells.^67^ Studies have also shown that the YAP signalling pathway interacts with other signalling pathways, such as VEGF, TGF‐β and Wnt, to regulate the development of PVR. Immunohistochemical analysis of VEGFR2 and YAP in human PVR membranes revealed an inverse correlation between the localization of VEGFR2‐positive and YAP‐positive cells.^68^ Zhang et al. showed that YAP activation increases with epidermal growth factor receptor (EGFR) activation and mediates progressive retinal fibrosis in mice.^69^ VEGFR and EGFR are essential for the development of PVR.^68^, ^69^ Blocking the YAP pathway also inhibits EMT in RPE cells and pro‐fibrotic responses in PVR induced by factors such as TGF‐β2^70^ and matrix stiffness.^71^ Meanwhile, a recent study by Lu et al.^25^ reported that in adult mouse RPE, loss of YAP activates the Wnt/β‐catenin pathway, leading to RPE cell dedifferentiation, loss of tight junctions, loss of RPE65 and pigments, and retraction of microvilli and basal infoldings, the typical features of EMT in RPE cells during PVR. Furthermore, changes in YAP/TAZ localization and CTGF protein expression in Müller cells may contribute to the development of PVR/retinal gliosis during retinal detachment.^72^

These findings reveal how the Hippo pathway and other signalling pathways interact to contribute to PVR development. Thus, targeting YAP/TAZ activity may be a potential therapy to slow the progression of PVR. More studies are needed to elucidate the mechanisms by which the Hippo pathway activation in the RPE inhibits PVR.

### 2.4 AMD

AMD is a leading cause of permanent blindness in the elderly population of developed countries. AMD is divided into two types based on its clinical and pathological characteristics: dry AMD, characterized by geographic atrophy (GA) and wet AMD, marked by choroidal neovascularization (CNV).^73^ In dry AMD, RPE cells undergo progressive degeneration, EMT and cell death, leading to GA.^74^ Wet AMD involves fibrovascular proliferation under the central retina.^75^ During CNV development, immature blood vessels in the choroid disrupt the Bruch's membrane and grow under the RPE, extending further into the subretinal space. Repeated leakage and haemorrhage from immature blood vessels inevitably lead to subretinal fibrosis and fibrovascular scarring, thereby damaging the retina.^76^ Wet AMD is responsible for most cases of central vision loss in AMD.^77^ The Hippo pathway has been identified as a key intracellular signalling system controlling angiogenesis.^78^ Therefore, current investigations of the role of the Hippo pathway in AMD have mainly focused on the treatment of late‐stage neovascularization and its accompanying fibrosis.

The Hippo pathway governs the proliferation and migration of ECs and is implicated in the pathogenesis of wet AMD. The results of pathway enrichment analysis revealed that in wet AMD patient samples (aqueous humour samples from 36 patients), six upregulated microRNAs regulate protein processing in the Hippo signalling pathway.^79^ According to recent studies, the photosensitizer verteporfin, which can inhibit YAP by increasing trypsin‐mediated cleavage of YAP,^80^ is used as a treatment for AMD.^81^ A recent study showed that YAP promotes ocular neovascularization by altering endothelial glycolysis, and intravitreal injection of small interfering RNA (siRNA) targeting YAP significantly inhibited neovascularization in a CNV mouse model.^49^ Another study revealed that YAP expression was upregulated after laser photocoagulation, and that YAP upregulation further promoted CNV formation by promoting EC proliferation in a mouse model of laser‐induced CNV.^82^

In addition to neovascularization, the Hippo signalling pathway regulates subretinal fibrosis and fibrovascular scar formation in AMD. The critical influence of YAP/TAZ on EMT^83^ and endothelial‐mesenchymal transition (EndMT)^84^ has been demonstrated in several earlier studies. Reduced nuclear retention of TAZ and ZEB1 may inhibit stress fibre formation in porcine RPE.^85^ Hypoxia increases YAP expression and activity in human umbilical vein endothelial cells (HUVECs).^75^ Recent studies have demonstrated that pharmacological inhibition or genetic depletion of YAP attenuates hypoxia‐ or TGF‐β2‐induced inflammation and EndMT in HUVECs, and reverses laser‐induced subretinal fibrotic changes in mouse models, providing a new therapeutic target for the treatment of subretinal fibrosis in wet AMD.^75^, ^86^, ^87^ Additionally, YAP is connected to the mitogen‐activated protein kinase (MAPK)/ERK pathway, participates in pericyte‐myofibroblast transition and promotes CNV fibrosis.^24^ In a laser‐induced CNV mouse model, YAP knockdown not only reduced the proliferation, migration and differentiation of human retinal microvascular pericytes but also attenuated pericyte‐myofibroblast transformation and subretinal fibrosis.^24^ These findings highlight the role of the Hippo pathway in AMD, and identify YAP/TAZ as possible targets for preventing AMD‐related neovascularization and subretinal fibrosis.

### 2.5 Glaucoma

Glaucoma is a primary cause of permanent blindness and is characterized by optic nerve injury and progressive retinal ganglion cell (RGC) degeneration.^88^ By evaluating the optic nerve head and peripheral parameters, glaucoma can be clinically staged as mild, moderate, or severe.^89^ The aqueous humour flowing through the trabecular meshwork (TM) is the primary site of intraocular pressure (IOP) regulation.^90^ Both abnormal fibrotic activity in human trabecular meshwork (HTM) cells and extracellular matrix (ECM) remodelling are responsible for increased aqueous humour outflow resistance and increased IOP.^91^, ^92^ Elevated IOP in rodent models produces optic neuropathology that was first observed in the unmyelinated portion of the optic nerve head, an area that corresponds to the lesion in human glaucoma.^93^ Gene expression patterns in the unmyelinated optic nerve showed significant enrichment in the Hippo pathway compared to that in the myelinated optic nerve and retina.^94^ The role of the Hippo signalling pathway in glaucoma is mainly reflected in the regulation of cell fibrosis and optic nerve protection.

Genome‐wide transcriptome analysis of HTM cells treated with TGF‐β1/2 showed that the Hippo signalling pathway is related to POAG and TM pathophysiology.^95^, ^96^ YAP/TAZ are present in the TM, and YAP functions in normal trabecular tissue, whereas TAZ has a major impact on glaucomatous trabecular tissue.^97^ Both matrix stiffness^91^ and composition^98^ can affect YAP/TAZ activation in HTM. YAP/TAZ can, in turn, regulate focal adhesion, ECM remodelling and the contractile properties of HTM cells.^91^ Yoo et al. found that the inactivation of YAP/TAZ attenuated HTM cell dysfunction in a tissue‐mimetic ECM microenvironment.^99^ Various factors, including Ras homologue gene family member A (RhoA),^100^ lysophosphatidic acid (LPA),^98^ and interleukin‐6 (IL‐6),^98^ can promote the expression and nuclear translocation of YAP/TAZ, thereby enhancing the fibrotic activity of HTM cells.^92^ Increased YAP/TAZ expression enhances HTM cell proliferation and actin‐associated protein activation, increases TM stiffness and impairs permeability in glucocorticoid‐induced glaucoma.^101^ Many factors, such as microRNAs (miRNAs),^102^ verteporfin,^103^ latrunculin‐B,^104^ and Wnt pathway activation,^105^ can inactivate YAP/TAZ, promote HTM cell proliferation, prevent cell damage, promote aqueous humour outflow and reduce IOP. These results indicated that YAP/TAZ plays a key role in the fibrotic activity of HTM cells.

In addition to HTM cells, YAP can promote LC cell fibrotic and proliferative phenotypes in response to stiffened LC present in glaucoma.^106^ Hippo signalling is also involved in mechanical strain‐induced glaucomatous scleral ECM remodelling. YAP binds to Smad3 and is involved in mechanical strain‐induced differentiation of human scleral fibroblasts.^107^ Furthermore, siRNA knockdown of YAP/TAZ partially reversed the transformation of human Tenon fibroblasts into myofibroblasts, which may inhibit fibrosis formation after trabeculectomy.^108^ Inhibition of YAP/TAZ has potential applications in the treatment of human conjunctival fibrosis after glaucoma surgery.^109^

In addition to fibrosis, YAP regulates the survival and function of associated cells, including RGCs and astrocytes. Disrupting the interaction between PITX2 and YAP impairs the transcription of multiple antioxidant genes, leading to elevated IOP and progressive RGC death.^110^ Astrocytic YAP can protect the optic nerve and retina. Conditional knockout of YAP in astrocytes results in severe inflammatory infiltration, optic nerve demyelination and RGC damage.^111^ Collectively, YAP/TAZ play a critical role in fibrotic activation and optic nerve protection during glaucoma development. These observations suggest that the Hippo pathway is required to maintain smooth outflow of aqueous humour and suppress elevated IOP and is closely related to glaucoma development and prognosis in glaucoma filtration surgery.

### 2.6 RB

RB arises from immature retinal cells and is the most prevalent intraocular malignancy in children under the age of six.^112^ Clinically, RB is classified as intraocular (stages A–E) or extraocular (stages 0–IV).^113^ It can be aggressive and life‐threatening if left untreated; however, it can be managed if it is identified early. However, many RB survivors become blind or lose their eyes. Therefore, it is necessary to identify new RB‐specific biomarkers, elucidate their molecular mechanisms and develop improved RB‐targeted therapeutic strategies.^114^ RB is caused by the biallelic deletion of the tumour suppressor gene RB transcriptional co‐repressor 1 (RB1) in 98% of cases, whereas 2% of cases have normal RB1 levels in tumours initiated by amplification of the MYCN oncogene.^115^

An increasing number of studies have shown that the Hippo pathway is important in RB.^114^, ^116^ Reduced LATS2 levels can strongly inhibit the induction of cellular senescence by RB protein.^117^ TAZ is highly expressed in RB tissues, and TAZ downregulation can inhibit retinoblastoma cell proliferation and block cell cycle progression.^118^ Clinical studies have found that high TAZ expression plays an important role in the aggressiveness of RB and predicts poor prognosis for RB patients.^118^ Similarly, another recent study highlighted the role of the YAP‐TEAD complex in RB, showing that disruption of the complex using verteporfin decreased cell growth and blocked cell cycle progression of RB cells.^26^ As cancer stem cells have been recognized as a source of cancer, RB stem‐like cells (RSLCs) have also been considered for RB therapy.^119^ Recent findings indicate that YAP/TAZ is essential for RSLC stemness, and the knockdown of YAP/TAZ drastically reduces the expression levels of RSLC markers.^120^ Therefore, YAP/TAZ inhibition may be an effective therapeutic strategy for treating RB in humans.

Song et al. revealed that miR‐224‐3p downregulation increases LATS2, activates the Hippo signalling pathway, and decreases YAP/TAZ expression, thereby promoting apoptosis, inhibiting RB cell proliferation and limiting tumour growth and angiogenesis.^121^ Over‐expression of miR‐125a‐5p, on the other hand, can significantly suppress tumour formation by suppressing TAZ expression in RB.^122^ By upregulating YAP and downregulating miR‐613, long noncoding RNA (lncRNA) can enhance RB aggressiveness and drug resistance.^123^ However, Pearson et al.^124^ showed that YAP/TAZ are undetectable/low in human RB tumours, and that upregulation of YAP/TAZ may prevent RB growth. Taken together, these findings suggest that the Hippo‐YAP pathway regulates RB cell proliferation and correlates with the prognosis of patients with RB. However, additional research into alterations in the expression and clinical therapeutic effects of YAP/TAZ in RB is still required.

### 2.7 UM

UM originates from extra‐dermal melanocytes in the uvea of the eye and is the most common primary intraocular malignant tumour in adults. It is characterized by insidious onset and poor prognosis.^125^ There are five stages of UM: stage 0 and stages I–IV, based on the TNM staging system (T, tumour; N, node and M, metastasis).^126^ Mutations likely to contribute to UM tumorigenesis occur primarily in two genes encoding the alpha subunits of G proteins, GNAQ^127^ and GNA11.^128^ The YAP/TAZ and MAPK pathways are the two major signalling pathways downstream of GNAQ/11 associated with UM.^129^ Studies have also found a higher activity of the YAP/TAZ pathway in UM tissues.^130^ Mutations in Gαq drive UM cell growth by activating YAP/TAZ. Gαq siRNA resulted in a nearly 50% reduction in YAP/TAZ nuclear localization in UM cells.^131^ Oncogenic GNAQ/11 can trigger the YAP pathway through Trio‐Rho/Rac‐focal adhesion kinase (FAK), leading to the dephosphorylation of YAP/TAZ and promoting its oncogenic activity, independent of the canonical Hippo pathway.^132^, ^133^, ^134^

Additionally, Lats1/2 kinases activation can enhance YAP phosphorylation and limit YAP nuclear accumulation, thereby specifically inhibiting UM tumour formation.^27^, ^135^ Direct inhibition of the YAP/TAZ‐TEAD interaction reduces cell viability in UM cell lines.^27^, ^136^, ^137^ Recent studies have also shown that activator protein 1 (AP‐1), a downstream effector of YAP, is required for YAP‐dependent UM growth driven by GNAQ/11 mutations, and that chemical inhibition of AP‐1 inhibits the growth of GNAQ/11mutant melanoma cells.^138^ Inhibiting the YAP/TAZ pathway using shRNA or medicines such as verteporfin,^139^ bromodomain and extra‐terminal inhibitors,^140^ dacarbazine^141^ and valproic acid,^137^ decreased UM cell proliferation in vitro and resulted in tumour regression in mice with a GNAQ/11 mutation.^132^, ^134^ Previous studies have shown that the Hippo/YAP signalling pathway can interact with other signalling pathways to regulate UM. Li et al.^27^ demonstrated that Lats1/2 deletion‐induced YAP/TAZ activation cooperates with the Ras/MAPK signalling pathway and promotes UM progression. Combined use of YAP and MALAT1 inhibitors can improve the therapeutic effects of UM.^142^ In addition, suppression of YAP/TAZ signalling can limit escape from MEK inhibitor therapy and improve therapeutic responses in UM, both in vitro and *in vivo*.^135^, ^143^ The combination of drugs that block both the YAP/TAZ and MEK/ERK signalling pathways can enhance the efficacy of UM treatment.^135^ Furthermore, histone deacetylase (HDAC) inhibition can block the increased output of YAP signalling and enhance the efficacy of MEK inhibitor therapy in UM.^144^ The combined inhibition of Mcl‐1 and YAP/TAZ is a potential therapeutic option for metastatic uveal melanoma.^136^

Therefore, by regulating the activation of the YAP/TAZ pathway, multiple factors can indirectly affect tumorigenicity and metastasis of UM cells. FAK inhibitors are already being tested in preclinical and early stage studies for their safety and effectiveness in UM (NCT04720417 and NCT04109456).^145^ Lactamase β overexpression attenuates the interaction of YAP with protein phosphatase‐1 catalytic subunit α, thereby increasing YAP phosphorylation, blocking YAP translocation to the nucleus, and inhibiting YAP activity, thereby inhibiting the tumorigenicity and metastasis of UM cells.^146^ Transcriptional interdependence between bromodomains and YAP has been identified in UM cells, and inhibitors can limit the growth and transmission of UM cells by reducing YAP expression.^140^ Taken together, these data indicate that YAP/TAZ could be a potentially useful target for UM therapy. Another study reported that UM‐specific survival was not significantly different between tumours with low and high YAP activity, and the survival of UM patients and cell lines was not significantly reduced by YAP/TAZ depletion.^147^ This study suggests that the effect of YAP on the development, growth and invasion of UM in patients may be lower than that demonstrated in experimental studies.^147^ Therefore, further studies are needed to determine the therapeutic effects of the components of the Hippo signalling pathway in UM.

## 3 CONCLUSIONS AND REMARKS

The Hippo pathway is an important regulator of organ size, cell proliferation, survival and differentiation during organ development and regeneration. The expression and subcellular localization of YAP and TEAD family transcription factors during mammalian ocular development have been well‐studied and summarized^135^, ^148^; however, the role of Hippo components in ocular pathogenesis is still poorly understood. Here, we summarize recent findings that provide evidence for the pivotal role of Hippo pathway elements in the pathophysiology of ocular diseases such as cataracts, AMD, DR, PVR, glaucoma, UM and RB. Although progress has been made in unravelling ocular disorders and the Hippo pathway, there are still many unsolved questions regarding the function of the Hippo pathway in ocular diseases.

Further research is needed to elucidate the answers to important questions, such as how the Hippo signalling pathway integrates with multiple regulatory networks that control ocular diseases and whether activation or inhibition of the Hippo signalling pathway can have a therapeutic effect on the disease. In addition, most studies on the Hippo signalling pathway in ophthalmic diseases have focused on YAP/TAZ while ignoring the role of other Hippo pathway components. Current studies mainly focus on cells or experimental animals and lack relevant clinical data. Furthermore, because the Hippo pathway can be regulated by RPE tight junction proteins, the role of YAP/TAZ in ocular immune privileges requires further investigation. A key limiting factor is our incomplete understanding of the pathophysiological function of the Hippo pathway. However, with a better understanding of the canonical and non‐canonical regulation of the Hippo pathway components in the ocular environment, we anticipate that treatments targeting the Hippo pathway will have beneficial therapeutic effects on ocular diseases.

## AUTHOR CONTRIBUTIONS

**Yuxiang Du:** Conceptualization (equal); data curation (equal); formal analysis (equal); resources (equal); supervision (equal); writing – original draft (equal); writing – review and editing (equal).

## CONFLICT OF INTEREST STATEMENT

The authors declare that they have no competing interests.

## Data Availability

The data that support the findings of this study are available from the corresponding author upon reasonable request.
